# Geography, altitude, agriculture, and hypoxia

**DOI:** 10.1093/plphys/kiae535

**Published:** 2024-10-04

**Authors:** Michael J Holdsworth, Huanhuan Liu, Simone Castellana, Mohamad Abbas, Jianquan Liu, Pierdomenico Perata

**Affiliations:** School of Biosciences, University of Nottingham, Loughborough LE12 5RD, UK; Key Laboratory for Bio-Resources and Eco-Environment, College of Life Science, Sichuan University, Chengdu 610065, China; PlantLab, Institute of Plant Sciences, Sant’Anna School of Advanced Studies, 56010 Pisa, Italy; Plant Stress Resilience group, Institute of Environmental Biology, Utrecht University, Utrecht 3541 TR, The Netherlands; Key Laboratory for Bio-Resources and Eco-Environment, College of Life Science, Sichuan University, Chengdu 610065, China; PlantLab, Institute of Plant Sciences, Sant’Anna School of Advanced Studies, 56010 Pisa, Italy

## Abstract

Reduced oxygen availability (hypoxia) represents a key plant abiotic stress in natural and agricultural systems, but conversely it is also an important component of normal growth and development. We review recent advances that demonstrate how genetic adaptations associated with hypoxia impact the known plant oxygen-sensing mechanism through the PLANT CYSTEINE OXIDASE N-degron pathway. Only 3 protein substrates of this pathway have been identified, and all adaptations identified to date are associated with the most important of these, the group VII ETHYLENE RESPONSE FACTOR transcription factors. We discuss how geography, altitude, and agriculture have all shaped molecular responses to hypoxia and how these responses have emerged at different taxonomic levels through the evolution of land plants. Understanding how ecological and agricultural genetic variation acts positively to enhance hypoxia tolerance will provide novel tools and concepts to improve the performance of crops in the face of increasing extreme flooding events.

## Introduction

With the escalation of global climate change and the increase in extreme weather events, food security faces serious threats. This highlights the urgent need to develop crops capable of withstanding harsh, rapidly changing environments, particularly those associated with reduced water availability (drought) or inundation (flooding). Understanding the genetic basis of natural variation in stress-related traits will not only enhance our understanding of plant environmental adaptation but also has significant implications for guiding crop breeding for stress tolerance. The model plant *Arabidopsis thaliana*, a member of the Brassicaceae family, exhibits a wide geographic distribution and genetic diversity, which equips it with robust adaptability to environmental shifts. For example, an analysis of flooding tolerance in 86 *A. thaliana* ecotypes revealed significant variation in tolerances (Vashisht et al. 2011), positioning this species as an exceptional model for studying plant responses to flooding stress. The discovery of an oxygen-sensing mechanism in *A. thaliana* (Gibbs et al. 2011; Licausi et al. 2011) through the PLANT CYSTEINE OXIDASE (PCO) N-degron pathway (Box 1) provided the framework to understand molecular responses to hypoxia and how genetic adaptation to tolerate low oxygen is linked to the perception of hypoxic environments either internally or externally (Box 2). In this context, we present recent advances in understanding the molecular mechanisms of adaptive evolution in plants related to oxygen sensing and signaling.

Box 1. **A molecular mechanism for plant hypoxia sensing**

**Figure 1.**
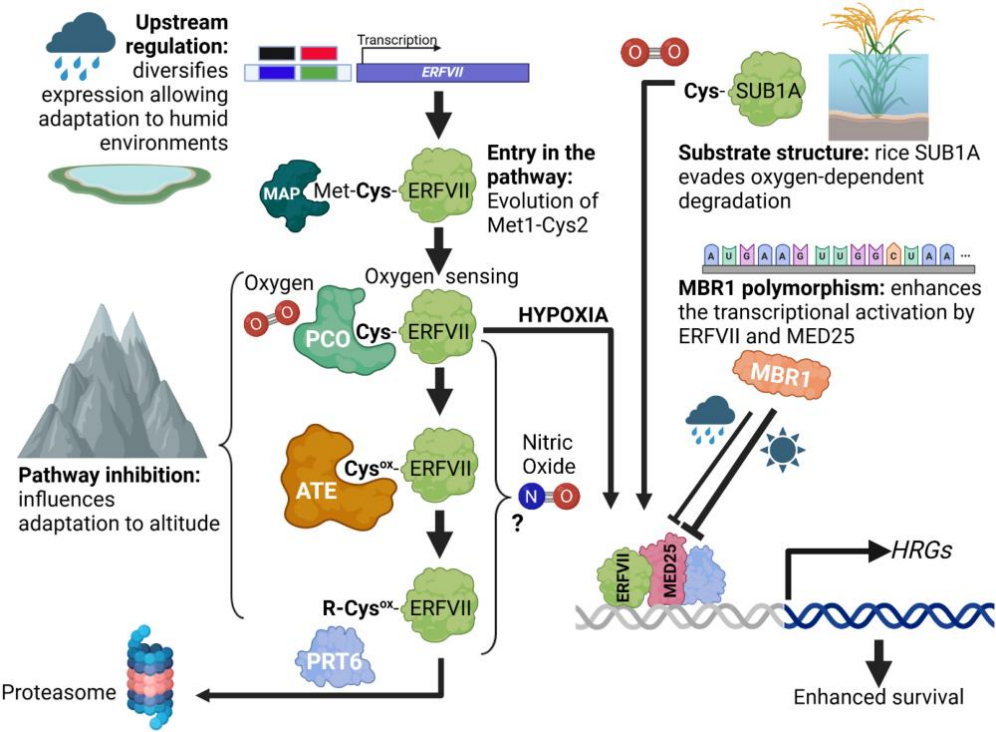
Genetic adaptation of the plant oxygen sensing system. The plant oxygen-sensing mechanism, illustrating genetic adaptions that have occurred through upstream regulation of gene expression, entry into the PCO N-degron pathway, inhibition of the pathway, and change in substrate structure to evade oxygen-regulated degradation. Positions of molecular oxygen and nitric oxide (NO) in the pathway are indicated. Unknown location of NO action indicated (?). Abbreviations: ATE, ARGINYL TRANSFERASE; HRPE, Hypoxia Responsive Promoter Element; MAP, METHIONINE AMINO PEPTIDASE; Met, methionine; PCO, PLANT CYSTEINE OXIDASE; PRT6, PROTEOLYSIS 6 E3 Ligase. CysOX indicates oxidized amino-terminal cysteine (sulfinic or sulfonic acid).

The only known mechanism of sensing reduced intracellular molecular oxygen (hypoxia) in plants is controlled through the PLANT CYSTEINE OXIDASE (PCO) branch of the PROTEOLYSIS (PRT)6 N-degron pathway (Fig. 1) (Holdsworth and Gibbs 2020; Holdsworth et al. 2020). This regulates the stability of proteins with amino-terminal Cys through the Ubiquitin Proteasome System (UPS). In the presence of sufficient oxygen PCO oxygen sensors oxidize amino-terminal Cys to Cys-sulfinic acid, that is a substrate for ARGINYL TRANSFERASE (ATE) amino-terminal arginylation (White et al. 2017). This in turn allows recognition of the modified amino terminus by the E3 ligase PRT6 and subsequent ubiquitylation and degradation (Zubrycka et al. 2023). The pathway relies on the low, physiologically relevant, affinity of PCOs for their substrates (Weits et al. 2014; White et al. 2018). Under hypoxia substrates accumulate because the action of PCOs on amino-terminal Cys is constrained, and following return to normoxia substrates are destabilized rapidly (Zubrycka et al. 2023). To date, 3 transcription-associated substrates of this pathway have been identified: group VII ETHYLENE RESPONSE FACTOR (ERFVII) (Gibbs et al. 2011; Licausi et al. 2011) and LITTLE ZIPPER2 (ZPR2) (Weits et al. 2019) transcription factors, and Polycomb Repressive Complex (PRC)2 component VERNALIZATION2 (VRN2) (Gibbs et al. 2018). Of these, ERFVIIs play the predominant role in responses to hypoxia, binding to the Hypoxia Response Element (HRPE), thereby activating expression of hypoxia-responsive genes, including those responsible for anaerobic metabolism (Zubrycka et al. 2023). In all cases these proteins initiate Met1-Cys2, and through the constitutive action of METHIONINE AMINO PEPTIDASE (MAP) amino-terminal Met is co-translationally removed to reveal Cys-2 (Zubrycka et al. 2023). Destabilization of substrates also requires nitric oxide (NO) (Gibbs et al. 2014), though the site of action of NO within the pathway has not been defined. This system is coordinated by the hormone ethylene that activates NO depletion, pre-adapting seedlings to low oxygen stress (Hartman et al. 2019), and is integrated into energy-sensing programs through the energy sensor TARGET OF RAPAMYCIN (TOR) (Kunkowska et al. 2023). A similar pathway exists in mammalian systems, mediated by the ADO oxygen sensor, to regulate reduced oxygen- and NO-controlled stabilization of a number of nontranscription-related proteins (Hu et al. 2005; Lee et al. 2005; Masson et al. 2019). Other potential oxygen-sensing systems have been suggested in plants though none have been experimentally validated (Holdsworth 2017).

Box 2. **Acute vs chronic hypoxia vs cyclic hypoxia**

**Figure 2.**
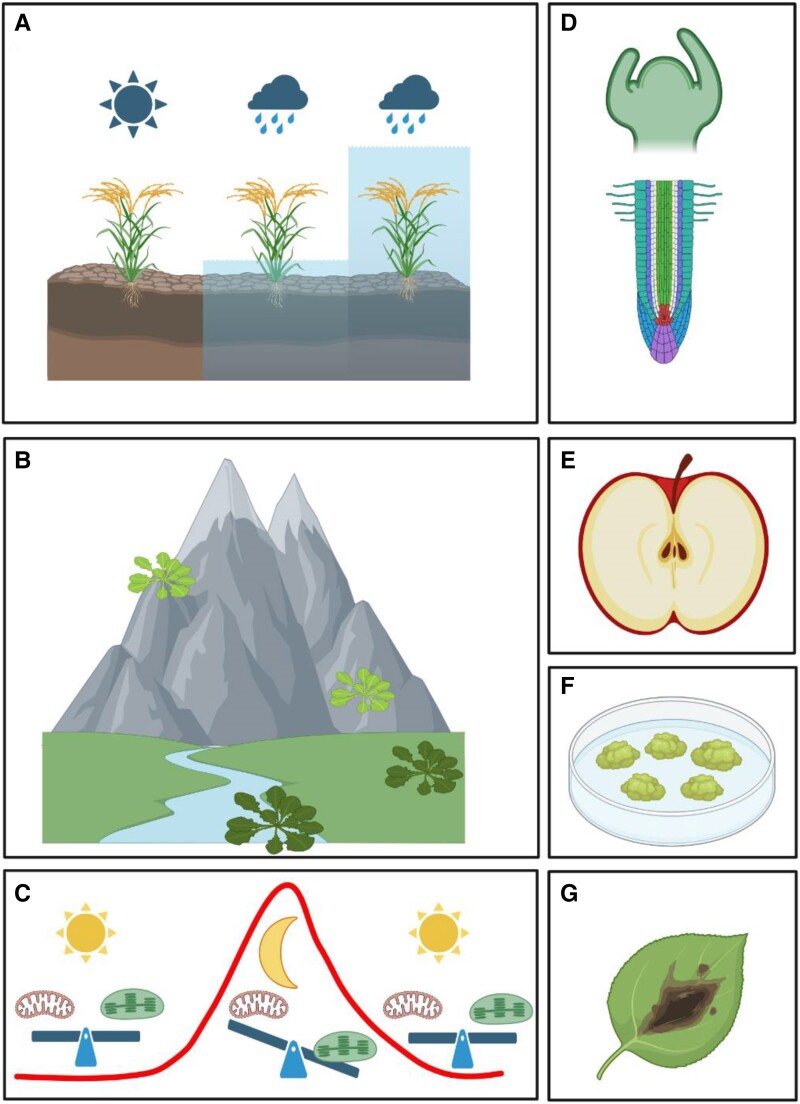
The many different types of hypoxia in plants. **A)** Hypoxia is one of the stresses to which plants are exposed after excessive rainfall if the soil becomes waterlogged and/or plants are partially or totally submerged. Only a limited number of plant species can tolerate prolonged submersion. **B)** Adaptation to hypoxia is needed to ensure fitness to different environments, for optimal growth at different ambient oxygen levels, such as for plants growing at different altitudes. **C)** Plants are exposed to fluctuating oxygen availability daily as the balance between oxygen production by photosynthesis and oxygen consumption by respiration changes diurnally. At night, young leaves are exposed to lower oxygen availability, that influences their growth rate. **D)** Hypoxic niches are constitutively present is some plant tissues, including root tissues, phloem, and the shoot apical meristem. In these cases, hypoxia does not represent a stress for the plant but rather a signal influencing growth and development. **E)** Bulky plant organs are often hypoxic, because oxygen diffusion is compromised. This has an impact, for example, on post-harvest fruit physiology (Xiao et al. 2024). **F)** Callus and in vitro grown plant tissues are often hypoxic, which may affect plant regeneration (Koo et al. 2024). **G)** Plant tissues affected by microbial growth can be hypoxic due to enhanced respiration in the infected area (Gravot et al. 2016; Valeri et al. 2021).

Plants are aerobic organisms and need oxygen for growth and development. Severely reduced ambient oxygen is incompatible with aerobic metabolism, a condition known as hypoxia. Hypoxia can be generated by an acute external event, such as excessive rain leading to complete or partial submergence, but also chronic, in hypoxic niches that exist in specific tissues (Fig. 2). Recently a third hypoxia type was discovered, cyclic hypoxia, that occurs diurnally in a spatiotemporal manner (Triozzi et al. 2024). During acute hypoxia, plants suffer a stressful condition as a result of adverse environmental conditions limiting oxygen availability. Soil waterlogging or complete plant submergence (that usually occurs in turbid water) following heavy rainfall are examples of acute hypoxia that limit oxygen availability. The frequency of extreme rainfall events, amplified by climate change, has risen globally (Hirabayashi et al. 2013; Blöschl et al. 2017), making acute hypoxia a severe challenge for agriculture. Actively maintained chronic hypoxic environments have been shown to be present in plants (Gravot et al. 2016; Kerpen et al. 2019; Weits et al. 2019; Valeri et al. 2021). Notably, the shoot apical meristem is hypoxic, and the gradients of oxygen likely serve as positional cues guiding leaf development (Weits et al. 2019). Additionally, physiologically chronic hypoxia is observed in other plant tissues such as lateral root primordia (Eysholdt-Derzso and Sauter 2017; Shukla et al. 2019) and anthers (Kelliher and Walbot 2012). Unlike hypoxia resulting from tissue thickness limiting oxygen diffusion, as seen in bulky fruits or tubers (Loreti and Perata 2020; Xiao et al. 2024), hypoxia in these tissues appears to be intentionally generated by a yet unknown process that may be related to the preservation of genome integrity in the germ line (Lane 2016). This results in a restriction in the presence of oxygen-sensitive proteins exclusively to these hypoxic niches that triggers oxygen sensitive developmental programs including ZPR2 (Weits et al. 2019) (involved in new leaf production) and VRN2 (Gibbs et al. 2018), involved in the transition to flowering. Cyclic hypoxia in plants was recently identified as a diurnal nocturnal reduction in oxygen levels within young emerging leaves during the night (Triozzi et al. 2024). This initiates a mild transient hypoxic response through stabilization of ERFVIIs and activation of downstream signaling, which is reset when photosynthetic oxygen production re-establishes normoxia during the day. Consequently, a metabolic shift from aerobic to hypoxic pathways occurs at night, fostering competition between aerobic and hypoxic metabolism. This adaptation allows the plant to sustain leaf growth based on carbon sources and the availability of oxygen.

## Geography: the role of local adaptation in shaping acclimatization to humid environments

Local adaptation in plants is a dynamic process shaped by geographical variation and environmental pressures (Kawecki and Ebert 2004). In addition to biotic influences, abiotic factors such as climate, soil composition, and topography are also primary drivers of local adaptation in plants (Forester et al. 2016) (Box 3). Geography is a key determinant, exerting significant influence by modulating the selective pressures experienced by plant populations across different regions (Leimu and Fischer 2008; Savolainen et al. 2013). As plants undergo local adaptation, they manifest distinct genetic and phenotypic variation reflecting specific local environmental conditions (Hereford 2009). Particularly noteworthy are plants inhabiting extreme environments, which boast a wealth of genetic diversity facilitating adaptation to challenging conditions (Hampe and Petit 2005; Lee-Yaw et al. 2016). Such plants serve as invaluable repositories of genetic resilience, housing traits that confer robustness against environmental stresses (Oh et al. 2012). The primary driver of acute hypoxic stress (Box 2) stems from flooding events, often associated with intense precipitation episodes (Loreti and Perata 2020; Jiménez et al. 2024) whose occurrences are increasing due to ongoing climate change (Hirabayashi et al. 2013; Tabari 2020). By examining individuals inhabiting high rainfall environments potential instances of local genetic adaptation to such conditions may be revealed, with implications for understanding underlying mechanisms of response to hypoxic stress in plants. Recent advances in genomic technologies, along with the growing availability of open source datasets for pedoclimatic data, have revolutionized the study of local adaptation (Advances Box); environmental genome-wide association studies (eGWAS) represent a powerful approach that leverages large-scale genomic and environmental datasets to identify candidate genes associated with specific environmental variables (Lasky et al. 2023). eGWAS can capture interactions between genotype and environment across diverse landscapes, providing a broad understanding of local adaptation. Previous research focused on *A. thaliana* adaptation to hot and arid environments (Exposito-Alonso et al. 2018), and precipitation dynamics have been somewhat neglected. Implementing this approach could significantly enhance our understanding of how genetic relationships influence plant adaptation to rainy environments, which in turn could provide valuable insights into how plants respond to hypoxic stress.

Box 3. **Definition of environmental/climate variables associated with acute hypoxia**

Environmental and climate variables play a pivotal role in shaping the occurrence of acute hypoxia, influencing oxygen availability in ecosystems (Geigenberger 2003). Factors such as waterlogging, flooding, and altitude significantly impact oxygen levels (Korner 2007; Bailey-Serres et al. 2012). Waterlogging and flooding events inundate terrestrial habitats, reducing oxygen availability (Voesenek et al. 2006). With ascending altitude, atmospheric pressure diminishes, consequently lowering partial pressure and therefore availability of oxygen (Korner 2007). Additionally, various abiotic stresses, including temperature extremes, soil salinity, and nutrient availability, contribute to the incidence of hypoxia. These stresses can indirectly affect oxygen availability by influencing ecosystem productivity and oxygen consumption rates (Greenway and Gibbs 2003). Recently, the emergence of big data science has revolutionized access to an expanding array of open-source datasets, notably including high-resolution geographical and pedoclimatic (soil type and climate conditions) data. Alongside this wealth of environmental information, an expansion in the scope of the collection of *A. thaliana* has occurred, with the plant being sampled across a diverse array of environments, including those characterized by extreme precipitation or altitude conditions (Alonso-Blanco et al. 2016). These resources hold great promise for advancing understanding of genome adaptation to hypoxia at the geographical scale. Pedoclimatic datasets furnish insights into precipitation patterns, soil attributes, and altitude variations, thus offering a broad overview of the environmental landscapes that can facilitate the onset of hypoxic conditions. By analyzing the responses of diverse plant populations to different pedoclimatic conditions, particularly through innovative methodologies such as eGWAS, it will be possible to unravel genetic and physiological mechanisms underpinning hypoxia tolerance (Lou et al. 2022; Castellana et al. 2024).

This could also shed light on the evolutionary mechanisms driving adaptation to waterlogged conditions, potentially identifying key genetic loci involved in anaerobic metabolism and stress signaling pathways. Such insights could pave the way for breeding programs aimed at enhancing crop resilience to climate change–induced fluctuations in precipitation patterns. Recently, Castellana et al. (2024) studied the interplay between genetic variation and environmental factors associated with geographical rainfall dynamics to uncover regulators of adaptation to hypoxic stress. Using publicly available datasets documenting rainfall patterns since the early 20th century (e.g. WorldClim, Climate Reserach Unit, Global Precipitation Climatology Centre), climate data were integrated with *A. thaliana* genomes compiled through the 1001 Genomes Project (Alonso-Blanco et al. 2016). This collection sampled accessions across diverse environments, capturing the geographic range of the species, including regions with contrasting levels of rainfall. eGWAS identified *MED25 BINDING RING-H2 PROTEIN 1* (*MBR1*) as a genetic determinant involved in adaptation to rainy environments. *MBR1* encodes an E3-ubiquitin-protein ligase, responsible for the regulation of MEDIATOR COMPLEX SUBUNIT (MED)25, a subunit of the MEDIATOR complex that conveys information from gene-specific regulatory proteins to the RNA polymerase II transcription machinery (Iñigo et al. 2012). MED25 has been shown to interact with VII ETHYLENE RESPONSE FACTOR (ERFVIIs) as part of the hypoxia response (Schippers et al. 2024). By acting as a bridge between RNA polymerase II and the ERFVIIs RAP2.2 and RAP2.12, MED25 enhances the hypoxic response, facilitating the induction of core anaerobic metabolism genes. Castellana et al. (2024) uncovered 2 nonsynonymous single nucleotide polymorphisms (SNPs) within the coding region of the MBR1 gene that occur together and are exclusively present in *A. thaliana* accessions inhabiting regions with high rainfall levels, particularly in Northern Europe. Accessions harboring these SNPs exhibited increased tolerance to an acute reduction in ambient oxygen compared with the reference accession Col-0 and demonstrated diminished capacity to degrade MED25, thereby enhancing its stability and promoting the activation of hypoxia-responsive genes. The association of these SNPs with hypoxia was reinforced through the identification of the same polymorphism in *MBR1* through eGWAS analysis using soil bulk density variables. This is important because soils with elevated bulk density exhibit a heightened propensity for water retention, creating conducive conditions for waterlogging (Manik et al. 2019). This sheds light on a novel component of plant adaptation to waterlogging, revealing the role of soil characteristics in shaping adaptive responses. The study demonstrated how an eGWAS approach can unveil novel components within molecular stress responses to increased water abstracted to different environmental conditions.

In addition to eGWAS, conventional GWAS based on Recombinant Inbred Lines (RILs) from different accessions are also effective in identifying key genetic variation associated with environmental adaptation. The *RAP2.12* gene was identified as a major quantitative trait locus associated with local adaptation for ecological shifts from drought to flooding tolerance (Lou et al. 2022). This study utilized a population of RILs derived from 2 accessions of *A. thaliana* collected in Sichuan and Tibet, which have, respectively, high and low humidity geography. Expression of the *RAP2.12* gene was induced in the Sichuan accession under flooding stress treatment but not in the Tibet accession and vice versa under drought stress treatment. Further investigation revealed that RAP2.12 enhances both flooding and drought tolerance in different *A. thaliana* accessions, through a modification in 2 core cis-elements, the WT and W boxes, in the *RAP2.12* promoter. The WRKY70 transcription factor (Guo et al. 2024) was identified as binding to the WT box cis-element in the *RAP2.12* promoter to enhance expression under submergence stress in populations distributed in humid regions, while other WRKY family transcription factors or unknown proteins enhance *RAP2.12* expression under drought stress in accessions derived from dry and arid regions. Geographical studies revealed that 660 accessions of *A. thaliana* that carry the WT box haplotype (hap) are predominantly found in humid regions, whereas only 12 accessions, 4 of which were highly sensitive to flooding (Vashisht et al. 2011), carry the hap-W box and are sparsely and narrowly distributed in drier regions. This indicates strong selection based on environmental water availability. Haplotype analysis and construction of a phylogenetic tree using *RAP2.12* promoter sequences revealed that the hap-W box is ancestral and present in closely related Brassicaceae species, while the hap-WT box originated after *A. thaliana* diverged from closely related species and expanded into humid regions worldwide. This study emphasizes the significant role of noncoding regions in generating innovative traits and highlights a regulatory mechanism whereby allelic variation in a cis-regulatory element can lead to increased expression of the same gene in different stressed populations. This study uncovered a novel genetic mechanism that enables the colonization of habitats from environments with contrasting abiotic stresses, suggesting that crop improvement could be achieved by manipulating combinations of cis-regulatory elements to create new crop types capable of withstanding multiple abiotic stresses. Flooding stress is a quantitative trait influenced by multiple genetic loci. In addition to the major gene *RAP2.12*, allelic variations in the promoter region of the *WRKY33* on chromosome 2 have also been found to play a crucial role in this process (Liu et al. 2024). The G variant of the *WRKY33* promoter impairs WRKY70 binding and activation under flooding stress, leading to reduced *WRKY33* expression and decreased flooding tolerance. Conversely, the C variant of the *WRKY33* promoter contains a complete WRKY70 binding site, which facilitates WRKY70 binding and enhances *WRKY33* expression under flooding stress (Liu et al. 2024). This, in turn, stimulates the expression of downstream hypoxia-responsive genes such as *RAP2.2* and improves flood tolerance. Reverse genetic studies can also contribute to the identification of key genes in the hypoxia signaling pathway that confer adaptation to humid environments through allelic variation. Guo et al. (Guo et al. 2024) discovered that the ACYL-CoA BINDING PROTEIN4 (ACBP4) interacts with WRKY70, positively regulating response to submergence stress. Variation at Ser638 plays a key role in regulating humid adaptation. The overexpression of the phosphorylated form of the ACBP4 protein at Ser638 significantly enhances tolerance to submergence stress. The haplotype hap-ACBP4-638Ser, which represents the functional form, is predominantly found in humid habitats, while the hap-ACBP4-638Ala, representing a nonfunctional form, is mainly present in dry and arid habitats. This allelic variation of ACBP4 is closely associated with the annual precipitation patterns of global *A. thaliana* accessions, likely facilitating environmental adaptation during the species' origin and subsequent expansion.

## Altitude: adaptation of the core oxygen-sensing pathway

Terrestrial altitude is characterized by complex environmental gradients, including temperature, solar radiation (including UV light), and precipitation, that occur at regional or local levels (Korner 2007). Temperature and light quality, in general, are subject to the altitude-for-latitude disparity through which gradients change with elevation and also distance from the equator (Jump et al. 2009). Thus, despite their importance in local genetic adaptation to altitude (Bomblies 2015), temperature and light do not act as cues for absolute altitude sensing globally.

The partial pressure of atmospheric gases, including oxygen (*pO_2_*), varies uniformly across altitude and could serve as a “true” environmental signal for altitude adaptation, and adaptation of animals for life at high altitude has involved selection of alleles of proteins in the animal oxygen-sensing Hypoxia Inducible Factor system (Witt and Huerta-Sanchez 2019). Studies of genetic adaptation in plant populations along elevation gradients have a long history (Méndez-Vigo et al. 2011; Montesinos-Navarro et al. 2011; Wingler et al. 2015; Zaborowska et al. 2021), but no molecular mechanism was linked to the sensing of absolute altitude. A recent study showed that one mechanism of plant adaptation to altitude is linked to natural variation in oxygen sensing and sensitivity via the PCO N-degron pathway (Fig. 1) (Abbas et al. 2022). Previous work investigating environmental and developmental hypoxia identified oxygen as an important ecological and positional cue during skotomorphogenesis (development of etiolated seedlings in the dark; characterized by an apical hook, elongated hypocotyl, and yellow cotyledons) (Abbas et al. 2015). Skotomorphogenesis is an adaptive developmental stage unique to angiosperms, associated with post-germination growth before soil emergence (Potuschak and Bachmair 2015). During skotomorphogenesis, angiosperms are unable to convert phototoxic protochlorophyllide (Pchlide) to chlorophyllide as part of the oxygen-requiring chlorophyll synthesis pathway. This is because they only possess a light-activated chloroplast enzyme for Pchlide reduction (Light-dependent NADPH-protochlorophyllide oxidoreductase [L-POR]) (Schoefs and Franck 2003). Abbas et al. (2022) showed that a mechanism for altitude adaptation through oxygen sensing in etiolated seedlings occurs via ERFVII repression of Pchlide accumulation. Whereas in *A. thaliana* accession Col-0 (originally collected at low altitude) etiolated seedlings increased Pchlide levels were strongly correlated with increased ambient oxygen, in the Col-0 *erfVII* mutant (lacking activity of all ERFVIIs) Pchlide accumulation was unaffected under different ambient oxygen levels. This indicates a requirement for oxygen sensing, modulated through ERFVIIs, to regulate Pchlide steady-state levels. ERFVIIs were shown to influence Pchlide levels by directly repressing transcription of enzymes of chlorophyll synthesis pathway (including CHL27 and L-PORs) but most importantly by enhancing expression of FLUORESCENT IN BLUE LIGHT (FLU), a key repressor of the first committed stage of chlorophyll synthesis (Meskauskiene et al. 2001). When assayed at sea level (a high ambient oxygen environment) accessions of diverse angiosperms (*A. thaliana*, *S. habrochaites*, and *B. distachyon*) originally collected at low altitude showed low levels of Pchlide, whereas accessions from higher altitude (adapted to a lower *pO_2_* environment) were hypersensitive, displaying higher Pchlide levels. However, in low ambient oxygen (equivalent to high altitude) this difference was abolished, and all accessions showed lower Pchlide levels. Introgression of the *A. thaliana prt6-1* E3 ligase mutation from the low-altitude accession Col-0 into the high-altitude accession Shahdara (Sha; originally collected at around 3400 meters above sea level in the Pamir mountains of Tajikistan) reverted the oxygen hypersensitive phenotype. Etiolated seedlings from reciprocal crosses between the Sha accession and the *erfVII* mutant (in Col-0 background) exhibited similar Pchlide levels to Col-0. Both results confirm that ERFVIIs are functional in Sha but genetically repressed at low altitude. In *A. thaliana* at high ambient oxygen, the stability of ERFVIIs was shown to be higher in low-altitude accession Col-0 than in high-altitude accession Sha, but ERFVIIs were strongly stabilized in both accessions at low ambient oxygen (Abbas et al. 2022; Zubrycka et al. 2023). These data indicated that low-altitude accessions contain an activity that represses the PCO N-degron pathway (that leads to increased ERFVII stability). This mechanism may function to match the level of phototoxic Pchlide to ambient oxygen so that at increasing altitude, equivalent phototoxic Pchlide levels are achieved in etiolated seedlings, allowing optimal establishment once seedlings emerge from under the soil surface and photomorphogenesis commences. In addition to chlorophyll synthesis, altitude adaptation through oxygen sensing was also shown to influence the expression of genes associated with metabolic response to hypoxia, including *ALCOHOL DEHYDROGENASE1* (*ADH1*), indicating that this mechanism has a general effect on gene expression through ERFVIIs.

## Agriculture: the roles of rice *SUB1A* and *SNORKEL* loci in improving tolerance to flooding

Asia stands out as a major rice (*Oryza sativa*) producing region, and South and Southeast Asia are characterized by unique monsoon climates. This can subject rice plants to the risk of partial or complete submergence due to flooding, having a severe impact on rice production. Diverse rice accessions have evolved unique mechanisms to cope with flooding stress, making them useful subjects for basic biological research. Currently, substantial research on flooding tolerance in rice primarily utilizes 2 types of genetic materials. The first is the flooding-tolerant indigenous cultivar FR13A, originating from South Asia. FR13A can withstand full submersion for approximately 2 weeks, referred to as “flash floods,” and can quickly resume normal growth once the floodwaters recede (Xu et al. 2006). During flooding, FR13A employs a “quiescent strategy” in which its internodes do not elongate but maintain a normal growth rate, enabling rapid recovery once the floodwaters recede (Xu et al. 2006). The key locus and allelic variations responsible for flooding tolerance in FR13A were identified and include 3 tandem genes encoding ERFVII-type proteins named Submergence *(Sub)1A*, *Sub1B*, and *Sub1C* located on chromosome 9. Further investigation revealed that *Sub1A* is the major effective gene, with 2 main alleles, *Sub1A-1* and *Sub1A-2*, found in rice. Sub1A-1 contains serine at position 186, which can be phosphorylated by MPK3 (Singh and Sinha 2016) and exhibits robust induction in response to flooding, making it crucial for flooding tolerance. In contrast, Sub1A-2 carries proline at site 186 blocking the phosphorylation by MPK3 (Singh and Sinha 2016) and lacks inducibility by flooding, rendering rice plants susceptible to submergence (Xu et al. 2006). Furthermore, it was shown that during flooding conditions, Sub1A-1 inhibits rice internode elongation by promoting the accumulation of SLR1 and SLRL1 (Fukao and Bailey-Serres 2008) while elevating *PYRUVATE DECARBOXYLASE* (*PDC*) and *ADH* levels to enhance anaerobic metabolism, consequently enhancing resilience to flooding. Moreover, Sub1A-1 can indirectly restrain the expression of *Sub1C* (Fukao et al. 2006; Xu et al. 2006), a pivotal regulator in stem elongation, thus impeding stem elongation during flooding. Interestingly, Sub1A-1 contains the classical ERFVII N-degron sequence MCGG but is not regulated through the PCO N-degron pathway (Gibbs et al. 2011) (Fig. 1). Further research revealed that the C terminus of Sub1A-1 can interact with its N terminus, sterically evading N-terminal degradation in this manner (Lin et al. 2019). Additionally, under flooding conditions, Sub1A-1 can directly bind to the promoters of 2 other ERFVII family genes, ERF66 and ERF67, forming a transcriptional regulatory cascade that enhances anaerobic metabolism and flooding tolerance when submerged (Lin et al. 2019). Unlike Sub1A-1, both ERF66 and ERF67 are correctly regulated by N-terminal degradation through the PCO N-degron pathway (Lin et al. 2019). It is noteworthy that many rice varieties, despite lacking the *SUB1A* locus entirely, still demonstrate remarkable flooding tolerance (Niroula et al. 2012; Samal et al. 2014; Okishio et al. 2015; Iftekharuddaula et al. 2016), indicating the existence of *SUB1A*-independent mechanisms that contribute to their response against flooding stress.

Another important genetic resource for studying flooding tolerance is deepwater rice, found in Southeast Asia, West Africa, and other regions (Hattori et al. 2011; Lin et al. 2023). Compared with the flooding-tolerant FR13A variety, deepwater rice exhibits superior resilience to prolonged deepwater floods, achieved through an “escape strategy” involving flood-initiated increased plant height. It is important to note that deepwater cultivated rice varieties, unlike flooding-tolerant varieties like FR13A, lack the ability to endure complete submersion and the subsequent oxygen deprivation stress (Mittal et al. 2022) as they can tolerate only partial submersion.

In response to submergence, internodes elongate rapidly to rise above the water surface, avoiding oxygen deficiency stress and enabling aerobic metabolism. Several major genes and their alleles responsible for flooding tolerance in deepwater rice have been successfully cloned and characterized. These include the *SEMIDWARF1* (*SD1*) gene (Kuroh et al. 2018) located on chromosome 1, the *ACCELERATOR OF INTERNODE ELONGATION1* (*ACE1*) gene (Nagai et al. 2020) on chromosome 3, and the *SNORKEL* (*SK*)*1* and *SK2* genes (Hattori et al. 2009) as well as the *DECELERATOR OF INTERNODE ELONGATION1*(*DEC1*) (Nagai et al. 2020) gene, all located on chromosome 12. Under flooding conditions, the phytohormone ethylene accumulates, inducing the core transcription factor ETHYLENE-INSENSITIVE3-LIKE (EIL)1a of the ethylene signaling pathway, which stimulates the expression of *SD1* and *SK1/2* genes, leading to increased levels of active gibberellins. This, in turn, stimulates internode elongation during deep flooding in deepwater rice. In non-deepwater rice varieties, the *SD1* gene is a null variant, and the *SK1*/*2* genes are absent (Hattori et al. 2009), limiting their ability to activate and accumulate gibberellins necessary for stimulating internode elongation (Kuroh et al. 2018). Furthermore, aside from the observed differences in internode elongation between deepwater and non-deepwater rice under shallow or deep flooding conditions, there are also significant distinctions in their responses to the application of exogenous gibberellin. This distinction derives from the following molecular mechanism: in deepwater rice, the proteins ACE1, which promotes internode elongation, and DEC1, which inhibits it, both respond to gibberellins. Under flooding or aging conditions, the *ACE1* allele in deepwater rice can respond to gibberellins to promote internode elongation. In non-deepwater rice, this allele is a null variant, resulting in the loss of its ability to respond to gibberellins and promote internode elongation during vegetative growth stages (Nagai et al. 2020). Interestingly, during the reproductive growth stage, both deepwater and non-deepwater rice adopt the same growth strategy. They can respond to gibberellins, promoting the expression of the *ACL1* (*ACE1-LIKE1*) gene, which shows the closest homology to *ACE1* among 6 *ACE1* paralogues and suppresses the expression of *DEC1*, thereby promoting internode elongation (Nagai et al. 2020). Further research has found that this mechanism of ACE1 promotion and DEC1 inhibition of internode elongation is conserved in the Poaceae family (Nagai et al. 2020). Deepwater rice can withstand flooding for months, but it is susceptible to oxygen-deprived conditions caused by complete submergence. On the other hand, FR13A can endure full submersion but only for around 2 weeks (Xu et al. 2006). Therefore, identifying key genes for flooding tolerance using unique rice germplasm resources holds significant theoretical and practical implications for achieving prolonged flooding tolerance. Breeding aimed at enhancing flooding tolerance in rice, drawing inspiration from the adaptive strategies of plants that thrive in flooded environments, is an ongoing endeavor.

## Evolution and adaptation to acute hypoxia

**Figure 3.**
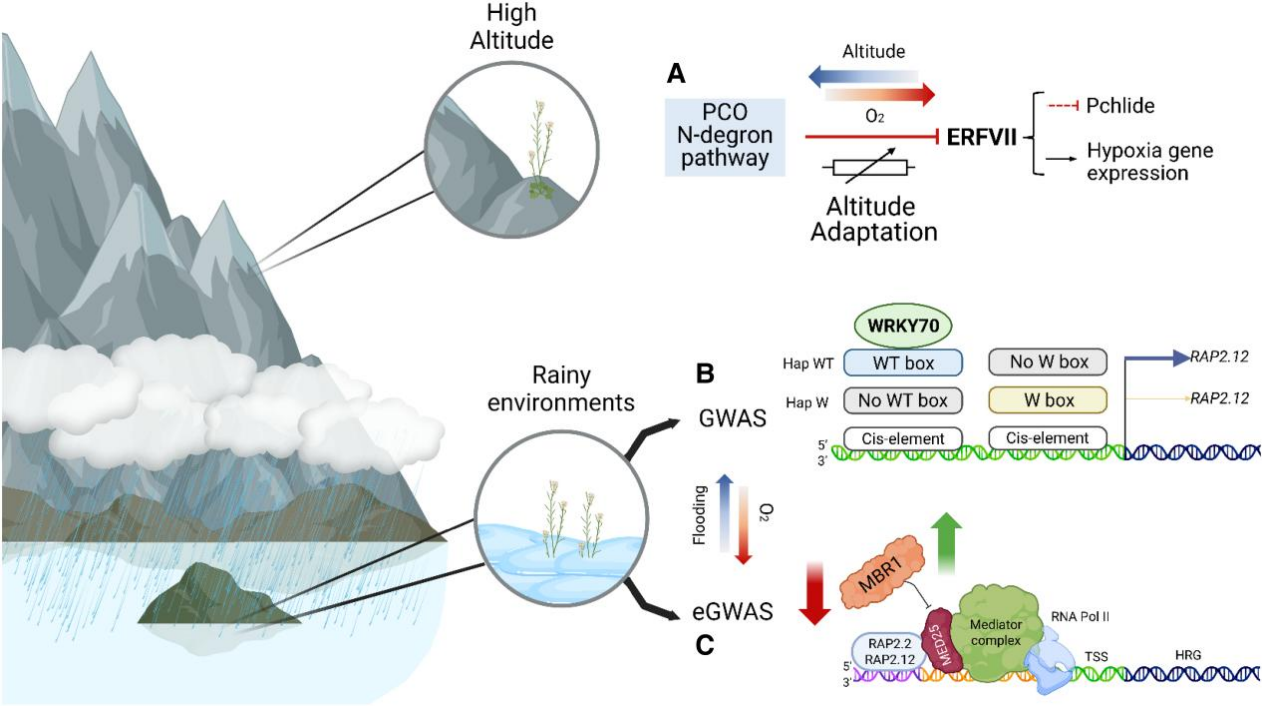
Geography/altitude adaptation: methodology and output. **A)** A model for altitude adaptation in angiosperms through oxygen sensing. Partial pressure of oxygen in inversely proportional to altitude. The arrow poiting right indicates decreasing oxygen availability with increasing altitude (arrow pointing left). Blocked arrows indicate repression. Altitude adaptation (rheostat symbol) occurs at the level of ERFVII stability, controlled via the PCO N-degron pathway, with high altitude plants showing increased sensitivity to oxygen. **B)** Allelic variations in the promoter region of the *RAP2.12* gene were found to be associated with geographic adaptation related to precipitation through GWAS analysis. Under flooding stress, *A. thaliana* accessions carrying the WT haplotype (WT box) in the promoter of *RAP2.12* can elevate its expression through the accumulated WRKY70 transcription factor, enhancing flooding tolerance. In contrast, *A. thaliana* accessions carrying the W haplotype (W box) in the promoter of *RAP2.12* cannot effectively elevate its expression and are therefore relatively sensitive to flooding stress. **C)** Model depicting the role of MBR1 in plant adaptation to rainy environments. Using an eGWAS approach, MBR1 was identified as a critical regulator in hypoxic response pathways. The MBR1 variant in rainy environments carries 2 non-synonymous SNPs, which impair its ability to degrade MED25 compared with the version found in non-rainy environments. This impairment results in MED25 being more stable, which enhances the activation of the ERFVII transcription factors given MED25 role as a regulator of gene expression. This facilitates an enhanced hypoxic response, providing plants with a survival advantage in waterlogged conditions. The arrow pointing down indicates highly rainy environments, where MBR1 stability is reduced; the arrow pointing upwards represents drier environments, where the protein remains more stable.

Recent advances in hypoxia research discussed in this update have greatly increased understanding of evolutionary processes related to hypoxia response at the molecular level, building on the very large body of previous work that provided an ecophysiological understanding of adaptations associated with acute hypoxia (Fig. 3) (Voesenek and Bailey-Serres 2013). Since the discovery of the PCO N-degron pathway as a mechanism for oxygen sensing, these advances can be placed in context with both the perception of oxygen (Fig. 1) and evolutionary trajectory. Whereas this pathway is deeply conserved in eukaryotes, substrates and associated mechanisms are not (Holdsworth and Gibbs 2020). Molecular variation associated with hypoxia signaling has occurred at many different stages in the evolution of land plants. For example, it was shown that hypoxia-inducible B-type PCO enzymes evolved only in spermatophytes (seed plants), which may result in increased efficiency in restraining fermentative metabolism (Weits et al. 2023). All substrates of the PCO N-degron pathway identified to date contain Cys-2, which is revealed following the activity of MAP (Fig. 1). It was shown that in VRN2, Cys-2 appeared only in angiosperms as a result of amino-terminal truncation of EMBRYONIC FLOWER 2 (EMF2) at an internal Met-Cys sequence evolutionarily fixed before divergence of angiosperms (Gibbs et al. 2018). This shows that entry of regulatory protein substrates into the pathway is one mechanism for controlling cellular processes in response to oxygen availability. Hypoxia-repression of Pchlide levels in etiolated seedlings was shown to be conserved in diverse lineages of angiosperms (Abbas et al. 2022). The angiosperm plastid genome, uniquely in land plant clades, has lost the *ChlB*, *ChlL*, and *ChlN* (light-independent protochlorophyllide reductase subunits) genes (Armstrong 1998). It is therefore likely that this trait, as a prerequisite for altitude adaptation, exists only in angiosperms. Diversification of promoter functionality through cis-element evolution in response to humidity occurred within *RAP2.12* at the species level. Similarly, nonsynonymous allele diversification in MBR1 and ACBP4, leading to altered protein function, also occurred following speciation in *A. thaliana*. Novel protein variants causing increased tolerance strategies to flooding (including Sub1A-1 and SK1/2) have evolved following domestication within different landraces of rice. These examples show that adaptation related to acute hypoxia is highly dynamic, indicating strong selective pressure to enhance survival under this stress, and suggests that many more examples remain to be discovered. It should be noted that in addition to oxygen sensing, the PCO N-degron pathway also senses nitric oxide and as a result influences tolerance to diverse abiotic stresses (Vicente et al. 2017; Lou et al. 2022), indicating that genetic adaptation to hypoxic stress may also be associated with increased tolerance to other stresses.

## Conclusions

Here we describe recent advances in understanding the relationship between the oxygen-sensing PCO N-degron pathway and selection in natural and agricultural settings for increased tolerance to low-oxygen environments. These developments have been achieved with the help of large collections of germplasm that has allowed georeferenced information to be associated with whole genome sequences (see Advances Box). Meaningful associations would not have been possible without a detailed understanding of the fundamental mechanism underlying oxygen sensing that has been delivered since the discovery of the biochemical framework of the PCO N-degron pathway firstly in animals and subsequently in plants (Lee et al. 2005; Hu et al. 2008; Gibbs et al. 2011; Licausi et al. 2011; Weits et al. 2014) (Box 1). These advances lay the groundwork for future work to investigate how the mechanism of acute hypoxia sensing is linked to the evolution of plant responses to humidity and ambient oxygen (see Outstanding Questions Box).

**ADVANCES BOX**

Diverse georeferenced germplasm resources with corresponding whole genome sequence data for both *A. thaliana* and rice: These provide resources for association studies linking genetic diversity, ecological adaptation, and evolutionary processes. They facilitate the integration of genomic and environmental data, enabling the exploration of complex plant environment interactions.

Open-source climatic datasets: high-resolution, freely accessible databases for climatic data represent a valuable source for understanding how organisms adapt to their environments and for unravelling the genetic mechanisms that allow species to thrive under diverse climatic conditions.

Online open platforms for GWAS: the development of online platforms for computationally intensive analyses like GWAS has facilitated access to high-throughput genomic analysis, making it more accessible and scalable for researchers.

**OUTSTANDING QUESTIONS BOX**

1. What is the pervasiveness of altitude adaptation through oxygen sensing? How do non-angiosperm plants directly sense altitude (do they?).
2. What is the nature of the PCO N-degron pathway repressing activity associated with altitude adaptation?
3. What are the mechanisms by which high-altitude crops adapt to the local environment?
4. Could mutations in MBR1 have evolved recently? Could this variant expand and become established in populations inhabiting rainy environments?
5. What other proxies could be used within eGWAS studies to associate plant populations with adaptation to hypoxia?
6. While all examples of oxygen-sensing adaptation so far discovered involve ERFVIIs, are other transducers of low oxygen (including ZPR2, VRN2, and as yet undiscovered Met1-Cys2 initiating proteins) also involved in adaptation either in natural or farmed environments?

## Data Availability

No new data were generated or analyzed in support of this update.

## Dive Curated Terms

The following phenotypic, genotypic, and functional terms are of significance to the work described in this paper:

WRKY70 Gramene: AT3G56400
WRKY70 Araport: AT3G56400

PRT6 Gramene: At5g02310
PRT6 Araport: At5g02310

WRKY33 Gramene: AT2G38470
WRKY33 Araport: AT2G38470

cysteine CHEBI: CHEBI:15356

methionine CHEBI: CHEBI:16811

SUB1A Gramene: DQ011598
SUB1A Araport: DQ011598
